# Authentic leadership, perceived organizational support, and psychological capital: Implications for job performance in the education sector

**DOI:** 10.3389/fpsyg.2022.1084963

**Published:** 2023-01-09

**Authors:** Uzma Sarwar, Muhammad Aamir, Yu Bichao, Zhongwen Chen

**Affiliations:** ^1^School of Education, Huanggang Normal University, Huanggang, China; ^2^School of Computer Science, Huanggang Normal University, Huanggang, China

**Keywords:** authentic leadership, perceived organizational support, psychological capital, job performance, education sector

## Abstract

The present study sifts the indirect role of psychological capital (PsyCap) in linking authentic leadership (AL) and job performance (JP). Furthermore, this study investigates the interplay of AL and perceived organizational support (POS) in PsyCap. We tested these assumptions through PROCESS macro with two sources of data collected from 350 employees and their respective colleagues working in education sector organizations in China. The study findings established that AL positively influences employee performance directly and indirectly through PsyCap. POS moderates the effects of AL on PsyCap such that this relationship gets more pronounced in individuals with high levels of POS. All organizations in the education sector can benefit from the current study’s practical application. We recommend that firms create and implement these training programs to improve JP since AL is favorably correlated with JP. The organization should pick executives with a vision to encourage e-JP. To promote this behavior, firms can also hold management training seminars, conferences, and programs. Making performance a clear necessity within job criteria will encourage it among personnel. To achieve great results, top management and leadership must inform the workforce about the importance of authentic behavior in the workplace.

## 1. Introduction

Quality leadership is desperately needed in the education industry, which functions in a complicated environment marked by technological and economic developments (Akhtar et al., 2021; Syed et al., 2021). As increasing cases of corporate fraud and scams are revealed throughout the world, the need for more positive, authentic, and value-based leadership has become more apparent (Akhtar et al., 2022). Stakeholders want their leaders to operate with a high level of honesty (Akhtar et al., 2020a). In this environment, genuine leadership has gotten a lot of press in the business world, prompting a spike in organizational behavior (OB) study (Akhtar et al., 2022). Authentic leadership (AL) is frequently regarded as a prerequisite for all other types of constructive leadership (Avolio and Gardner, 2005). AL has enhanced self-awareness *via* an ethical climate and transparent relationship with followers (Walumbwa et al., 2008). Self-awareness relates to one’s perception of oneself, the degree to which one is aware of one’s strengths and faults, and one’s effect on others. An internalized moral perspective, in which one’s actions and behavior are regulated by personal values and moral principles, is a metaphor for self-regulation. The level of openness and honesty with which one shares information and expresses one’s actual emotions is referred to as relational transparency. The degree to which a leader weighs all relevant information before making a choice is referred to as balanced processing. The possibility of regaining stakeholder confidence, trust, hope, resilience, and optimism is highlighted by supporters of AL (Avolio et al., 2004).

The research on AL is still in its early stages, which is surprising given the novelty of the idea. Despite the conceptualization shared by academics and practitioners that AL fosters encouraging employee behavior at work, there is a dearth of actual evidence to support this claim (Gardner et al., 2021). AL, such as moral integrity, care for others, and consistency between actions and moral principles, attempts to create a productive workplace (Clapp-Smith et al., 2009; Hannah et al., 2011). Leaders are moral and genuine. Since the concept is new, AL research is still in its very infant stage. Although theorists and practitioners alike contend that genuine leadership fosters positive employee attitudes and behavior in firms, there is a dearth of empirical data to support this claim (Gardner et al., 2011). Despite the benefits of AL on employee behavior, there are currently few empirical studies that support the relationships and mechanisms between AL and followers’ behavior (Ribeiro et al., 2020). Haque et al. (2021) found that the mechanism through which leaders stimulate followers’ behavior has not been well analyzed. Further research is still needed to substantiate these claims, according to Ribeiro et al. (2020), to confirm the AL-behavior linkage needed to identify the relevant mediating factors. Kim et al. (2022) stated that AL boosts task performance *via* emotional sharing. Aria et al. (2019) confirmed that AL affect turnover intentions *via* POS among teachers. Moreover, it is not well understood how genuine leadership affects follower results psychologically from the ground up (Lei et al., 2021; Akhtar et al., 2022). Although recent research has identified self-efficacy as a potential mediator (Lei et al., 2021) of the link between AL and employee outcomes, investigating various underlying mechanisms allows for a deeper understanding of the nature of interactions. This is especially true for a developing concept like AL, which is still in its early phases of growth. AL is considered a part of human resource management (HRM). Recently, studies have focused on the outcomes of HRM, such as job performance (JP) (Akhtar et al., 2020a; Yu et al., 2022), social capital and performance (Singh et al., 2021), psychological safety (Moin et al., 2021), and presenteeism (Haque, 2018).

Since human energy can be infectious, encounters between staff members and AL might help them recharge (Wang and Xie, 2020). Psychological capital (PsyCap) includes features such as self-efficacy, optimism, hope, and resilience. An AL energizes and motivates his or her subordinates while also bringing rich resources into the organization under the signaling theory. Under authentic leaders, employees’ PsyCap, hope, and positive affectivity grow (Luthans and Avolio, 2003; Rego et al., 2014). PsyCap was found to be favorably associated with the majority of employee behavioral outcomes (Luthans and Youssef-Morgan, 2017). The AL style holds that leaders develop their legitimacy *via* moral underpinnings, respect, and open communication with their followers. Typically, AL encourages openness and facilitates the growth of trust between superiors and followers, both of which are essential for personal success (Walumbwa et al., 2008).

There have been no empirical studies in OB research too far that examine the link between AL and PsyCap and their association with individual performance. Furthermore, limited empirical data indicate how cognitive processes may explain the association between AL and individual performance. The lack of clarity on the nexus between these two critical variables and the methods *via* which AL relates to individual performance hinders the ability of OB researchers to provide evidence-based recommendations. Given the positive impact of PsyCap witnessed in OB and the role of AL recognized by extant research in positive psychology to achieve personal success (Luthans et al., 2019). The primary aim of this study is to determine whether individual PsyCap mediates the effects of AL on their performance at various levels of perceived organizational support (POS).

The moderating function of POS is also investigated in this study. In addition, POS can forecast employee outcomes when combined with other factors (Chen and Eyoun, 2021; Côté et al., 2021). Côté et al. (2021) stated that POS buffered the presenteeism and job satisfaction relationship in the education sector. Employees with higher POS experience decreased turnover intentions (Huang et al., 2021) and job stress (Xu and Yang, 2021). Higher POS workers are more prone to have a sense of entitlement (Alnaimi and Rjoub, 2021). As a result, in the context of education, the current study uses POS as a moderator between AL and PsyCap.

The current study makes a noteworthy contribution to the literature on AL. To the best of our knowledge, this is the first study to look at the connection between AL and worker performance using PsyCap. This study, therefore, contributes to the corpus of research that has already examined the impacts of AL, which in turn contributes to the consequences of AL. Second, we think the relationship between AL and PsyCap is more complicated and susceptible to external organizational influences. Thus, we add to the corpus of current information by proposing POS as a boundary condition on the AL–PsyCap relationship. By reexamining human resource policy, this study offers managers a comprehensive framework for encouraging AL in the workplace.

## 2. Theory and hypotheses development

The signaling theory explains the information asymmetries between two parties, by which asymmetric information is disturbed by the quality of information about intent (Stiglitz, 2000). When employees receive signals, such as moral integrity, care for others, and consistency between actions and moral principles, they attempt to create a productive workplace from AL, so individuals can feel PsyCap, and they are actively indulged in positive outcomes.

### 2.1. AL and job performance

Nearly six decades ago, the notion of AL was first discussed in the literature, resulting in a rapidly expanding body of academic and empirical study (Gardner et al., 2011). Since the theory’s birth, AL has been considered one of the most important components affecting followers’ behavior (Wang and Xie, 2020; Gardner et al., 2021; Akhtar et al., 2022). According to Akhtar et al. (2022), leaders who lead individuals with authenticity can promote a favorable atmosphere and long-term follower accomplishment. When AL fully acknowledges their talents and weaknesses, communicates their thoughts honestly, maintains an appropriate proportion in their perspective, and exhibits strong moral values in their conduct and interactions, an increase in JP is expected (Alilyyani et al., 2018). AL is viewed as the primary source of good leadership behavior needed to achieve constructive work-related results (Avolio and Gardner, 2005).

Authentic leadership acts according to its ideals and works to establish open and sincere communication with its followers (Gardner et al., 2005). It may set an example for others and practice open communication (Avolio and Gardner, 2005). By setting an example for others, leaders exhibit their dedication to the task at hand and instruct those who follow them on how to be mentally, physically, and emotionally alert while working. According to Walumbwa et al. (2010), real leaders’ ethical actions are likely to influence their followers because of their allure and authority as role models. Under AL, followers often absorb the beliefs and values of the leaders and act in a way that is congruent with their ideals and ethics. For instance, AL is seen by followers as being governed by high moral standards and characterized by fairness, honesty, and integrity in interacting with followers (Avolio et al., 2004). Because of their openness, optimism, and high ethical standards, these leaders can inspire others to share their ideals. As a result, followers are inspired to engage in constructive action and develop a sense of self-worth and duty to return the favor (Ilies et al., 2005).

Authentic leadership sets a good example in the workplace by demonstrating confidence, hope, and optimism (Gardner et al., 2005). Positive attitudes and emotions may be infectious, leading to a good trickle-down effect within companies to stimulate positive emotional and cognitive growth among their followers, resulting in more JP. AL examine all necessary facts objectively while making a choice; they provide a fair and transparent workplace atmosphere. Employees in such a workplace are more conscious of the value of assisting others and are encouraged to do so (Walumbwa et al., 2010); hence, they participate in high JP. Therefore, we argued that:

**H1**: AL is positively related to JP.

### 2.2. The mediating role of psychological capital

PsyCap (hope, optimism, self-efficacy, and resilience) of any individual has a great impact on the JP of that specific individual. On the other hand, AL could also create such a wonderful and achievable environment in which they can perform in a better way because AL develops such a capacity under which they put their optimal efforts that are more beneficial to achieve the desired goals of himself. Therefore, when leaders adopt the AL style, they will prepare authentic followers who will grow with the qualities of AL (transparency, moral/ethics, balanced processing, and self-conscience). It is criticized that the environment in which AL adopts that permit an undefended atmosphere under which access of followers could be enhanced for support, information, and resources (Walumbwa et al., 2008).

AL develops a capacity in the followers under which they can complete their work efficiently and effectively (Akhtar et al., 2022). It enables them to perform efficiently and settle/compromise with the job and be emotionally strong; this way, the AL cultivates positive emotions in the followers (Bento and Ribeiro, 2013). PsyCap also plays a vital role in the behavioral and mental development of a person. Previous research has found that employees’ PsyCap is critical to the effective completion of their jobs (Luthans and Youssef, 2004). Employees with more PsyCap, according to Karatepe and Karadas (2015), are frequently active and committed, and they like being absorbed in their job (Peláez Zuberbühler et al., 2021).

Psychologically capable individuals have strong self-efficacy and confidence in their abilities; as a result, they can completely participate in their job, which may allow them to satisfy JP beliefs (Xanthopoulou et al., 2007). Furthermore, they are resilient, which means that even if they fail, they can recover and re-engage in their task, allowing them to constantly spend their energy (Bakker et al., 2011). They are also optimistic; as a result, they stay involved and do not stray from their route of completing the task at hand, fully pouring their resources into the work and performing effectively (Syed et al., 2021).

**H2**: PsyCap mediates the relationship between AL and JP.

### 2.3. Moderating role of POS

Previous studies concluded that when employees get developmental training and pay raises, they feel obligated and repay the organization by achieving its objectives (Eisenberger et al., 2002; Rhoades and Eisenberger, 2002). POS is viewed as a resource in an organization that produces psychological outcomes (Xu et al., 2022). People perceive how they are treated by the organization through their superiors, which results in a perception of the amount of power they believe is being exerted over them (Aselage and Eisenberger, 2003). In the education sector, POS is positively related to pro-unethical work behavior (Wang X. et al., 2021), work engagement (Karatepe et al., 2022), and commitment (Zagenczyk et al., 2021). Regarding the impact of POS on PsyCap, Yang et al. (2020) concluded that organizational support predicts PsyCap. Existing studies stated that POS significantly moderates and mediates organizational relationships (Naseer and Raja, 2021).

In the education sector, individuals need POS to PsyCap to serve their customers. As in the education sector, POS works as a social clue that attenuates the RL–PsyCap relationship. We contend that POS reflects an individual’s belief about his organization (Eisenberger et al., 1986), individuals with a high level of POS will optimize the salience of AL and increases the likelihood that employees experience PsyCap. In the case of high POS, employees perceive that they get an extra reward, praise, and recognition against JP, so they can feel happy (Eisenberger et al., 2020), thus making them more prone to perform their job with vitality. POS facilitates vitality at work as, due to POS, individuals feel obligated and want to repay the organization by exerting high efforts for goal achievement (Eisenberger et al., 2002). POS promotes learning at work since it encourages people to learn new things and develop their abilities to assist the organization in achieving its objectives.

**FIGURE 1:**
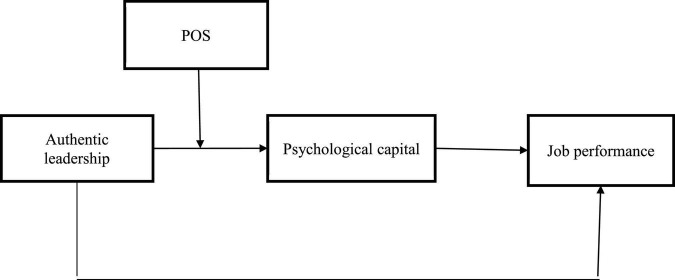
Hypothesized framework.

This personification tendency assigns organizations’ human-like individualities (Eisenberger et al., 1986). According to Salancik and Pfeffer (1978), high trust, integrity, fair distribution of rewards, praise, and recognition work as social clues, and thus, individuals actively engage in PsyCap. AL provides these particular resources for PsyCap. Alternatively, individuals with low POS levels view their organization has little or no commitment toward them. Then individuals with low POS are likely to perform their work with low vitality and learning. Akhtar et al. (2019) study how employees who experience high levels of POS feel obligated to enhance their JP to meet company goals and do it with all of their hearts. Thus, high POS will amplify the AL–PsyCap relationship (see Figure 1).

**H3**: POS moderates the AL–PsyCap relationship in that the higher the POS, the stronger the aforementioned relationship.

## 3. Research method

Cross-sectional data from the education sector organization were used in the current study. Data were collected from January 2022 to February 2022. Individuals are the unit of analysis. We delivered 550 survey forms containing questions about AL, POS, and PsyCap, as well as demographic information, to respondents, and we got 410 usable answers. In addition, we circulated JP’s survey form to the respondents’ peers and got 372 useful responses. We gathered 350 legitimate responses after removing 22 responses that had multiple or inadequate answers (e.g., two replies to a single statement). The following requirements had to be met by employees: they had to be a part of a certain team or department, have a direct leader within that team or department, and operate in an environment where there was unity and constant communication between team members. Participation was optional, anonymous, and based on written informed permission with a flexible withdrawal policy. In addition, the researcher collected the surveys right after they were completed. We started by outlining the goal of the investigation. The study team emailed the management of each firm in advance of the survey’s administration to describe in detail the study’s goals (i.e., AL and its effects) and its methodology. Following the official’s clearance, the same study polled each supervisor (by email), working with the heads of their respective departments, to describe the nature and objectives of the current study and inquire about their openness to having their followers.

To reduce frequent method biases, the current study implemented procedural remedies and recommendations made by Podsakoff et al. (2007). We started by outlining the goal of the investigation. The study team emailed the management of each firm in advance of the surveys administration to describe in detail the study’s goals (i.e., AL and its effects) and its methodology. Following the official clearance, the same study polled each supervisor (by email), working with the heads of their respective departments, to describe the nature and objectives of the current study and inquire about their openness to having their students participate. In addition, the poll asked supervisors to inform their staff of the study’s objectives and methods and seek their participation.

Based on prior research, which indicates that having support first from the top improves the responsiveness of the prospective respondents, we chose this strategy (Dillman, 2000). Second, data were collected through a multi-source design. By which the surveys of AL, POS, and PsyCap were tapped among respondents along with their demographic details. Furthermore, the survey of JP was tabbed among the peers of the aforementioned respondents. Third, in the present study, we also used a moderator to minimize the CMB (Simons and Peterson, 2000). Previous studies reported that the chances of CMB were minimized in the presence of a moderator (Javed et al., 2020, 2021; Syed et al., 2020, 2021; Aslam et al., 2021; Li et al., 2022).

### 3.1. Measures

All the variables were measured by previously validated scales. All the items will be measured by the use of a five-point Likert scale.

A total of 16 items AL questionnaire (ALQ) used to measure AL Avolio (2007)α is 0.97. The item is *My supervisor admits if his/her decision was wrong/mistaken*. We measured POS with the help of the eight-items scale developed by Eisenberger et al. (1986). A sample question was, “My organization considers my goals and values.” The α is 0.91. The 12 items of PsyCap were adapted from Luthans et al. (2007). An example scale is “I am confident that I could deal efficiently with unexpected events.” The α was 0.92. We utilized a peer-reported seven-item scale created by Williams and Anderson (1991) with a reliability score of 0.92 to measure JP. The example items include “*he or she adequately completes assigned duties*.” Recently studies used the peers rating scale to measure JP by which peers rated their colleague’s performance (Akhtar et al., 2021; Syed et al., 2021).

From a total of 350 responses, 234 were men (with 66.9%) and 116 were women (with 33.1%). Likewise, 35.7% are less than 29 years old, which shows that most of our respondents are young. In total of 27% respondents are 39 years old, 22% respondents are 49 years old and 15% respondents are 50 and above years old; 18% of respondents are at the graduate level, 70.9% of respondents have master’s degrees, and 11.1% of respondents are MS degree holders. Notably, 43.4% of respondents have less than 5 years of experience, 25.7% of respondents have up to 10 years of experience, and 30% of respondents have more than 10 years of experience.

As some potential variables may influence thriving at work and megaphoning behavior, therefore, gender, age, education, department, and experience were treated as control variables because previous studies also controlled them for PsyCap and JP.

## 4. Results

### 4.1. Measurement model

All the study variables’ reliability, convergent validity, and discriminant validity were calculated in the current study. The findings stated that estimated loadings of all items of each underlying construct are statistically significant and greater than the 0.4 threshold (see Table 1; Hair et al., 2010). The measures are reliable as the value of Cronbach’s alpha is greater than the cutoff level of 0.7 (Nunnally and Bernstein, 1978; Nunnally, 1994). We employed average variance extracted (AVE) and composite reliability (CR) to measure internal validity (Fornell and Larcker, 1981). All variables have CRs larger than 0.7 (Nunnally and Bernstein, 1994), and the AVE of each construct is greater than the cutoff level of 0.5 advocated by Fornell and Larcker (1981). Furthermore, we utilized the Fornell and Larcker (1981) approach to verify discriminant validity since the correlation between the two associated variables was >for each √ of AVE (see Table 2).

**TABLE 1:** Confirmatory factor analysis: validity and reliability.

Latent variables	Standardized loadings	AVE	CR	Alpha
AL		0.70	0.974	0.97
Item 1	0.712			
Item 2	0.710			
Item 3	0.839			
Item 4	0.804			
Item 5	0.838			
Item 6	0.924			
Item 7	0.774			
Item 8	0.832			
Item 9	0.887			
Item 10	0.696			
Item 11	0.895			
Item 12	0.850			
Item 13	0.856			
Item 14	0.924			
Item 15	0.896			
Item 16	0.898			
POS		0.574	0.909	0.91
Item 1	0.505			
Item 2	0.964			
Item 3	0.511			
Item 4	0.535			
Item 5	0.892			
Item 6	0.859			
Item 7	0.592			
Item 8	0.998			
PsyCap		0.510	0.922	0.92
Item 1	0.592			
Item 2	0.617			
Item 3	0.669			
Item 4	0.574			
Item 5	0.739			
Item 6	0.981			
Item 7	0.533			
Item 8	0.944			
Item 9	0.664			
Item 10	0.944			
Item 11	0.508			
Item 12	0.623			
JP		0.633	0.920	0.92
Item 1	0.675			
Item 2	0.573			
Item 3	0.985			
Item 4	0.619			
Item 5	0.941			
Item 6	0.944			
Item 7	0.717			

All loadings are significant at the 0.01 level. AVE, average variance extracted; CR, composite reliability.

**TABLE 2:** Discriminant validity test results.

Latent constructs	1	2	3	4
1. AL	0.837			
2. POS	0.367	0.758		
3. PsyCap	0.635	0.102	0.714	
4. JP	0.416	0.395	0.402	0.795

The √ of the average variance extracted was shown on the diagonal.

### 4.2. Correlation

According to Table 3, AL has a positive and significant correlation to POS (*r* = 0.53, *p* < 0.01), PsyCap (*r* = 0.65, *p* < 0.01), and employee JP (*r* = 0.63, *p* < 0.01). POS shows the positive and significant correlation to PsyCap (*r* = 0.47, *p* < 0.01) and employee JP (*r* = 0.52, *p* < 0.01). PsyCap shows the positive and significant correlation to the employee JP (*r* = 0.64, *p* < 0.01).

**TABLE 3:** Correlations mean and standard deviation of study variables.

Sr ^#^		Mean	SD		2	3	4
1	AL	3.29	1.30	**(0.94)**			
2	POS	2.85	1.24	0.53**	**(0.93)**		
3	PsyCap	3.11	1.0	0.65**	0.47**	**(0.90)**	
4	JP	3.48	0.91	0.63**	0.52**	0.64**	**(0.83)**

*N* = 350. **p < 0.01. ^#^Means number. Bold values means are significant.

### 4.3. Hypotheses testing

Table 4 displays the findings for our H1, H2, and H3 as direct and indirect hypotheses. We employed PROCESS macro (model 4) to examine this indirect effect and PROCESS macro model 1 to assess the moderating effect (Hayes, 2017) PROCESS macro (model 4), and to test the moderating effect, we used the model 1 of PROCESS macro. With the help of PROCESS macro, we also calculated the bias-corrected confidence interval by using the bootstrap technique. The current study also employs control variables, that is, age, education, and experience, but control variables did not significantly alter the main findings of the study. Thus, for clarity and parsimony, we excluded them from tables (Carlson and Wu, 2012); however, results with controls are available from the authors.

**TABLE 4:** Mediating role of PsyCap.

Sr. no	Variable	*R* ^2^	B	SE	*T*	*P*
1	AL → JP	0.60	0.77	0.06	12.83	0.000
2	AL → PsyCap	0.60	0.72	0.07	10.28	0.000
3	Psychological capital → JP	0.51	0.76	0.06	12.67	0.000
**Bootstrap results for indirect effects**						
			M	SE	LL 95% CI	UL 95% CI
Effect	Psychological capital		0.31	0.06	0.10	0.37
**Indirect effect and significance using normal distribution**						
			Effect	SE	Z	*P*
	Sobel test		0.31	0.06	5.17	0.001

*N* = 350. Control variables: marital status, department, and grade. The boot (LLCI and ULCI) from bias-corrected bootstrapping test. **p < 0.01, ***p < 0.001.

SE, standard error.

For H1, we anticipated that AL is positively related to JP. Results are shown in Table 4 and support our H1, as AL had a positive and significant association with positive JP (β = 0.77, *p* < 0.001). Furthermore, H2 states that AL is positively related to JP *via* PsyCap. In Table 2, we also present the indirect effect estimate (from 10,000 bootstrap samples) of AL on JP *via* PsyCap with 95% confidence intervals. Our results reveal that the indirect effects of AL on JP *via* PsyCap were significant (β = 0.31, boot 95% CI [0.10, 0.37], did not include zero). Providing support to our H2.

**FIGURE 2:**
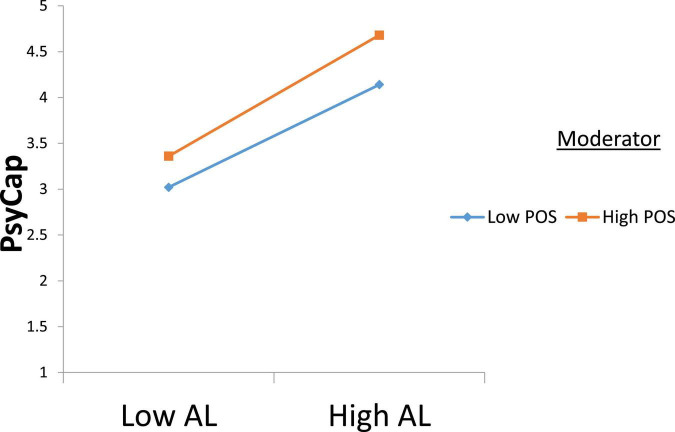
Interaction plot.

H3 observed the moderating role of POS on the association between AL and PsyCap. Table 5 demonstrates that AL and POS have positive and significant interactive effects on the PsyCap (β = 0.05, SE = 0.02, *p* < 0.05). The interactive effect pattern provides support to our H3. By following the suggestion of Aiken et al. (1991), we also demonstrated the characteristics of the interaction term. Figure 2 demonstrates that slop analysis provides support to our H3, by indicating that the interactive effect was stronger at a high level of POS (β = 0.45, boot 95% CI [0.22, 0.69], did not include zero) and weaker at a low level of POS (β = 0.20, boot 95% CI [0.03, 0.37], did not include zero). Providing support to our H3.

**TABLE 5:** Moderating role of POS.

Predictors	PsyCap			
	***R* ^2^**	**Estimate**	**SE**	**Sig**
AL		0.61	0.35	0.08
POS		0.22	0.11	0.04
AL × POS	0.11	0.05	0.02	0.02
**Conditional direct effects of X on Y at values of moderator**				
**POS**	**Effect**	**Boot SE**	**LLCI**	**ULCI**
−1 SD	0.20	0.08	0.03	0.37
*M*	0.33	0.08	0.16	0.49
+ 1 SD	0.45	0.12	0.22	0.69

*N* = 350. *p < 0.05, **p < 0.01.

## 5. Discussion

In this study, we created and evaluated a conceptual model that describes how AL affects employee JP. Two ideas were conceptualized for the investigation. The original model proposed PsyCap as a mediator of the AL–JP link. POS was proposed in the second model to moderate the aforementioned link. Both ideas were supported by the findings. AL was discovered to affect JP both directly and indirectly through PsyCap. High AL employees felt their work had a purpose and had greater JP than lower AL employees. It was discovered that the PsyCap fostered by sincere leaders helped people think and perform successfully. The psychological condition of having meaningful experiences increased intrinsic motivation and caused workers to give everything they had (May et al., 2004). The active employees felt the need to articulate themselves in ways to enhance their JP. They were passionate and prepared to go above and beyond in their job to improve their JP.

Employees with high PsyCap have the strength and energy essential to perform well (Choi et al., 2020), and their more frequent experience of good affect (Fredrickson, 2001) compelled them to seek unusual paths and perform better (Choi et al., 2020). This supports our claim that AL improves perceptions of PsyCap by requiring workers to engage in decision-making and sustaining a trusting relationship through relational transparency. A person’s confidence in his capacity to generate creative ideas and partake in creative activities is increased by this sense of worth and value. In addition, feeling acknowledged increases one’s psychological readiness to do vocational duties (Kahn, 1990). The findings add and extend the findings of previous studies on AL, demonstrating its link to PsyCap (McDowell et al., 2018; Ramalu and Janadari, 2020; Wang D. et al., 2021) and JP (Duarte et al., 2021). The findings highlight the importance of AL in education sector firms in cultivating good employee behavior. These findings from education sector firms are especially significant since AL is an emerging construct, and proof of its good consequences from many cultures is required on the path toward robust theory development. This study, therefore, contributes to the body of knowledge and aids in the advancement of AL by offering crucial empirical data on its effects in a distinctive cultural context.

The findings indicate that POS modifies the relationship between AL and PsyCap. People are more likely to gain greater PsyCap from AL when they perceive that their organizations are supporting them. Previous scholars have also investigated the moderating impact of POS (Côté et al., 2021). Côté et al. (2021) concluded that POS buffered presenteeism and job satisfaction relationship. Kumar et al. (2022) confirmed that POS moderates the employee capital and taking charge behavior. As a result, the findings of their study agree with those of the current study. POS thereby modifies the association between AL and PsyCap, which is direct.

### 5.1. Theoretical implications

First, this study expanded the body of study on the impact of AL with findings (Gardner et al., 2021). Even though enough studies show the favorable effects of AL on employee results, there are not many studies that show how AL affects long-term, fruitful relations with employees (Gardner et al., 2011). The study’s results also suggested that a brief bond, such as PsyCap, may act as a mediator between AL and JP.

The moderation research model is the second contribution of this study. The present study found that the relationship between AL and PsyCap was moderated by POS. By using POS as a moderator, the majority of earlier studies analyzed and examined the likelihood that AL and POS will interact either synergistically or substitutively. However, our study aims to address Iqbal et al. (2018)’s need for researchers to look at how AL employs behavioral outcomes. According to our research, AL actively encourages JP among its personnel. In particular, their interplay prompts us to reconsider the notion that researchers should pay attention to both the AL and organizational variables that are closer to the AL, such as POS, in addition to just the AL itself.

### 5.2. Practical implications

The finding has important ramifications for organizational managers as well. The managers must be authentic. According to the findings of this study, AL affects the PsyCap of subordinates. Managers generally push their frontline staff to offer exceptional education by either empowering or supporting them (Johanson and Woods, 2008) or raising their incentives, rewards, or acknowledgment. Managers may enhance their employees’ emotional control by being more real when engaging with education staff since authenticity reduces work strain and encourages work resources (Walumbwa et al., 2008). Therefore, leaders in the education sector would work to become more authentic in their relationships with others by asking for input, stating what they mean, owning up to their mistakes when they are incorrect, and basing choices on their core values (Gardner et al., 2005).

The findings offer valuable insights to top management in terms of improving JP. The findings show that AL improves JP by increasing PsyCap. AL will assist organizations in developing an active staff, committed, and engaged in their work, in addition to assisting them in establishing such a workforce. Keeping employees motivated has evolved as the most challenging HR issue facing firms due to the expansion of multinationals in the education sector that are all vying for the same talent. Our study advances knowledge on the factors influencing PsyCap. The study gives an essential tool to organizational managers for fostering a JP-oriented workforce by establishing AL as a forecaster of PsyCap and JP.

### 5.3. Limitations and future directions

The limitations and future directions of this study are listed below. The orientations of the links between the variables in this study may shift, decrease, or even be strengthened by some of the factors as a result of AL’s influence on JP *via* PsyCap. Other potential processes explaining the benefits of AL on JP deserve additional study and exploration. In the education sector, for example, job autonomy and feedback may be linked to AL and JP. Second, the relationships between AL and subordinate JP should be investigated in diverse education situations. Employees may develop or adopt various tactics for dealing with demands in various circumstances (Seymour, 2000). Third, additional studies on leaders’ motivating functions in the education sector should be done. The energy of followers may be affected differently by different leadership philosophies, either increasing or decreasing it. This has been determined to be essential to each person’s JP (Baum and Youngblood, 1975). Positive leaders, like spiritual and caring ones, give their followers energy, while negative ones, like oppressive and destructive ones, drain it from their followers. In light of this, the results of other styles of leadership, especially dark leadership such as despotic leadership (Syed et al., 2020), leaders’ knowledge hiding (Akhtar et al., 2021), exploitative leadership (Syed et al., 2021), and super ostracism (Akhtar et al., 2020b), on the JP of education employees should be investigated from the viewpoint of human power.

## 6. Conclusion

The subject of how AL affects public sector employees’ JP in the unique setting of China was expanded upon in this study. The results of the investigation support the viability of the proposed concept, which explains how AL raises JP among employees. In addition, PsyCap plays a crucial role as a mediator and advances the friendship between AL and JP. The results of this study also suggested that POS mediated the association between AL and PsyCap. Therefore, this research will enable them to understand the importance of AL, and its application across all industries will become essential for the JP of employees.

## Data availability statement

The raw data supporting the conclusions of this article will be made available by the authors, without undue reservation.

## Ethics statement

The studies involving human participants were reviewed and approved by the Ethics Committee of Huanggang Normal University. The patients/participants provided their written informed consent to participate in this study.

## Author contributions

US works on initial model, theory, literature review, and Research method. MA works on Introduction, literature review, and Research method. YB works on data analysis. ZC works on discussion and proof reading. All authors contributed to the article and approved the submitted version.
